# Commentary: The Implementation of China's Mental Health Law-Defined Risk Criteria for Involuntary Admission: A National Cross-Sectional Study of Involuntarily Hospitalized Patients

**DOI:** 10.3389/fpsyt.2019.00121

**Published:** 2019-03-12

**Authors:** Huajian Ma, Yang Shao

**Affiliations:** Shanghai Mental Health Center, Shanghai Jiao Tong University School of Medicine, Shanghai, China

**Keywords:** risk criteria, definition, involuntary admission, China, Mental Health Law

Having read your Brief Research Report entitled “The Implementation of China's Mental Health Law-Defined Risk Criteria for Involuntary Admission: A National Cross-Sectional Study of Involuntarily Hospitalized Patients” (Nov. 06, 2018) (1), we have some alternate opinions.

The article did put forth some interesting findings, but we do not agree with the conclusion that only 45.3% of involuntary admissions in China meet the Mental Health Law (MHL)-defined criteria. We believe that the reasons behind this result should be interpreted differently. In this article, the authors defined “MHL-defined risk criteria” as “an attack on others/themselves or endangering public security or impulsive aggression,” which is too narrow of a definition and does not reflect the original meaning of the legislation.

Actually, the risk criteria of involuntary admission (article 30) in the Mental Health Law, although has no official English version, refers not only to an obvious behavior, but also to a possibility. For example, Chen and his colleagues translated this criterion as (2): “*Inpatient treatment of mental disorders shall generally be voluntary. If the result of the psychiatric evaluation indicates that a person has a severe mental disorder, the medical facility may impose inpatient treatment if the individual meets one of the following conditions:* ① *self-harm in the immediate past or current risk of self-harm;* ② *behavior that harmed others or endangered the safety of others in the immediate past or current risk to the safety of others*.” In other translated version (3) “risk” was translated as: “① *having committed any act of harming himself or herself or having the potential to harm himself or herself; or having committed any act of endangering the safety of others or* ② *having the potential to endanger the safety of others*.” Despite the differences in translation, whether “*current risk*” or “*the potential*” is far broader than the author's definition.

Different translated versions of risk criteria reflect fuzziness of the definition to some extent (4, 5), thus the author's interpretation of risk criteria might be different from that of those psychiatrists'. According to our recent survey of 332 psychiatrists in China (published in Chinese), most participants considered impairment in self-care ability a risk of self-harm and threatening speech a risk to the safety of others (6). Thus, it is more appropriate to describe Jiang's finding as those 45.3% of patients being admitted because of obvious harmful behavior. At the same time, the remaining cases cannot be simply judged to be inconsistent with the MHL-defined criteria. They may be mixed with various situations, some of which were at risk of harming oneself or others to some extent and others might be quite inconsistent regarding legal requirements. Further analysis of medical records is needed to distinguish between these situations. Of course, Jiang et al. may have followed the correct definition but failed to communicate their definition adequately due to misleading terminology, which further explanation by Jiang et al. might be needed to make it clear.

The vague definition of risk criteria in MHL was the result of a compromise between the civil liberties approach, which highlights the importance of individual freedom and autonomy, and the medical model, which emphasizes the need for treatment as a sufficient prerequisite for involuntary admission. On the one hand, a narrow and strict definition of dangerousness may lead to delayed treatment (7). On the other hand, expansion of the criteria used to determine dangerousness could intensify the public perception of individuals with mental disorders as “dangerous,” and thus exacerbate the stigma and discrimination of persons with mental disorders (8). The final choice of legislation reflects the difficulty faced by the government in balancing the benefits to society and the individual and in transforming the delivery of mental health services (4).

Of course, the author's concerns that some mental illness patients' civil rights might have been violated in China are not alarmist. There is no clear definition of “current risk” in the criterion for involuntary admission, which may open a loophole for abuse of this clause (5). Psychiatrists, family members, and lawyers representing the patient may have different understandings of the coverage of such clauses and practices or may litigate on the basis of what they believe to be the *status quo* (9).

To make things even worse, the review mechanism for involuntary admission in MHL can only be used for a patient who is admitted because of “risk to others,” so if someone was sent to a psychiatry hospital with the reason being “risk to self,” he/she is unable to lodge a complaint (10). Such ambiguous legal definition of risk criteria, along with the lack of an effective independent involuntary admission review mechanism, makes it difficult to achieve the goal of protecting the rights of the mental illness patient.

Some recent research has also noted this problem. In our survey, 54.5% of respondents thought that somebody with a history of attacking others can be considered dangerous to others, and 33.1% of respondents thought that stopping taking medicine was a danger to the patients themselves (6). These findings were similar to our previous research before the MHL was enacted (9), which highlights the difficulties for psychiatrists to immediately change their attitudes according to the law reforms. Another study found that psychiatrists inappropriately encourage families to produce evidence of a patient's behavior that is harmful to one's self or others in order to legally commit the patient (11). These findings not only imply that change caused by law reformation usually comes in stages but also show that psychiatrists are more concerned with a patient's rights to receive treatment than the right of autonomy. Thus, there is still a long way to go in protecting the rights of people diagnosed with mental illnesses.

In order to effectively protect the rights of mental health patients, the following measures should be taken. First, more comprehensive and practical guidelines should be developed for psychiatrists to strengthen the protection of mentally ill patients' rights vis-à-vis the processes of admission. Furthermore, an independent review mechanism regarding the appeal of all involuntary admission cases should be set up. Besides, involuntary admission might be needed to a lower degree if service use and the availability of treatment resources for severe psychiatric patients could be improved in other ways without further increasing stigmatization hopefully (12).

## Author Contributions

HM wrote the main draft of the manuscript. YS critically revised the draft of the manuscript and edited the final version.

### Conflict of Interest Statement

The authors declare that the research was conducted in the absence of any commercial or financial relationships that could be construed as a potential conflict of interest.
